# Diurnal variation of inflammatory plasma proteins involved in pain

**DOI:** 10.1097/PR9.0000000000000776

**Published:** 2019-08-13

**Authors:** Hajer Jasim, Anders Carlsson, Björn Gerdle, Malin Ernberg, Bijar Ghafouri

**Affiliations:** a Division of Oral Diagnostics and Rehabilitation, Department of Dental Medicine, Karolinska Institutet, Scandinavian Center for Orofacial Neuroscience (SCON), Huddinge, Sweden; b Department of Medical and Health Sciences, Pain and Rehabilitation Centre, Linköping University, Linköping, Sweden

**Keywords:** Diurnal, Mass spectrometry, Nanoliquid chromatography, Plasma, Proteomics

## Abstract

**Introduction:**

Proteomics is a powerful approach for biochemical research because it directly studies the main functional components of biochemical systems. The understanding of the normal fluctuations of the proteome in health is essential to identify pain-specific biomarkers.

**Objective:**

To investigate fluctuations of the plasma proteome in healthy pain-free individuals.

**Methods:**

Blood samples were structurally collected in the early morning and evening from 10 clinically healthy individuals (26.3 ± 3.3 years). High abundant proteins were removed from plasma, and proteins were then analysed by nanoliquid chromatography combined with mass spectrometry. In addition, an assay of 71 cytokines/chemokines/growth factors was analysed.

**Results:**

Multivariate statistical analysis displayed that there were up to 64 proteins whose expression levels were significantly altered between the plasma samples collected during the morning and evening; no changes existed for the assay. The levels of 34 proteins were increased and 30 proteins were decreased during the evening compared with the morning sample. The increased proteins were involved in the biological processes such as protein activation cascade, complement activation, and stress response. The decreased proteins were involved in regulation of endopeptidase activity, inflammatory response, and protein metabolic processes.

**Conclusion:**

The circadian variations in the plasma proteome stress the need to collect blood samples of both patients and controls at a fixed time of the day. The results in this study might be useful for better understanding of the complexity of individual variation in the human plasma proteome over time and provide a baseline for improved pain biomarker discovery.

## Abstract 2

Supplemental Digital Content is Available in the Text.

## 1. Introduction

Plasma proteomics have gained much interest to identify biomarkers of different pain conditions.^11,36^ Proteomics is a powerful approach for biochemical research because it directly studies the main functional components of biochemical systems, the proteins. Because plasma is in direct contact with all organs and tissues, changes in body function are likely to be reflected in the plasma composition, and subsequently quantitative changes in the plasma proteome could thus serve as biomarkers of systemic effects.^10^ When the proteins expressed within a sample have been properly identified, they will then become a powerful tool to study global changes in proteins levels and expression under different conditions.^33^

Numerous proteomic studies have been successfully performed in different painful condition, eg, ischemic heart disease,^5,29^ lower back pain,^39^ cancer,^13,33^ rheumatoid arthritis,^40^ fibromyalgia,^9,27^ endometriosis,^7,15^ osteonecrosis,^20^ and osteoarthritis^4^ by analyzing proteins in blood. Due to practical clinical circumstances, blood samples used in studies are often drawn at different time points for different patients. The circadian clock, which regulates the biological rhythms, is usually disregarded.^21,32^ The circadian rhythmic secretion of several biomarkers related to pain and inflammation has been demonstrated.^3,12,16,18,22,26,37^ The core clockwork mechanism in the suprachiasmatic nucleus, located anteriorly of the hypothalamus gland, has been proposed to modulate the rhythmic expression.^3,18,26^

Due to the diurnal variations of several single biomolecules in plasma, it seems important to consider the fluctuations of the plasma proteome. To the best of our knowledge, no previous studies have investigated the individual plasma proteome and cytokine/chemokine/growth factor changes over time in well-characterized healthy individuals. Hence, the aim of this study was to investigate the fluctuations of the plasma proteome in healthy pain-free young adults using mass spectrometry–based proteomics combined with multiplex analysis of low abundant inflammatory proteins.

## 2. Materials and methods

### 2.1. Participants

Ten healthy participants, 5 men and 5 age-matched women, with a mean ± SD age of 26.3 ± 3.3 years were recruited though advertisement to participate in the study. The mean body mass index of the participants was 21.5 ± 3.0 kg/m^2^ and they were all nonsmokers. None reported infections, immune or neuropsychiatric disorders, high blood pressure, current pain, and pregnancy or lactation. None used any medications, contraceptives, or dietary supplement. Participants who reported elevated levels of psychological distress were excluded from further involvement in the study. All participants underwent a careful clinical examination and were asked to fill-in validated questionnaires as described below.

All participants received information regarding the objectives and procedures of the study and provided their informed written consent before the start of the study. The study was approved by the Regional Ethical Review Board in Stockholm, Sweden (2014/17-31/3), and followed the guidelines according to the Declaration of Helsinki.

### 2.2. Protocol

Participants were evaluated by the Swedish version of the Diagnostic Criteria for Temporomandibular Disorders (DC/TMD) axis I and axis II.^30^ The evidence-based protocol was used as a screening instrument for identification of participants with TMD signs that may not be presented during the interview and general examination. Participants showing clinical signs of TMD were excluded from further involvement in the study.

The following brief screening instruments included in the DC/TMD axis II questionnaire^30^ were used to measure symptoms of depression, somatization, and anxiety: the Patient Health Questionnaire (PHQ-9 and PHQ-15) and the Generalized Anxiety Disorder scale (GAD-7). In addition, the Perceived Stress Scale-10 (PSS-10) assessed stress levels.^23^

To investigate variation in the plasma proteome, venous blood samples were collected in the early morning at 7:30 am and in the evening at 7:30 pm from all subjects. The morning sample was collected, after overnight fasting, from the cubital vein into 8.5-mL EDTA PPT tubes (Vacutainer PPT, Becton, Dickinson and Company (BD), Franklin Lakes, NJ), and, subsequently, blood sampling was repeated in the evening at 7:30 pm The samples were mixed gently for 1 minute and then immediately placed on ice. Within half an hour, the samples were centrifuged at 1000*g* for 15 minutes at 4°C, and the upper 2/3 of the plasma was stored as aliquots at −70°C until analysis.

### 2.3. Proteomic analysis

Briefly, 40 µL of the plasma sample from each subject was depleted of albumin and immunoglobulin G using ProteoPrep (Sigma-Aldrich Co, St Louis, MO) according to the user manual followed by protein concentration measurement using 2D-Quant Kit (GE Healthcare, Little Chalfont, United Kingdom). Reproducibility analysis was performed on a plasma sample from a healthy participant. The sample was depleted, desalted, and concentrated in triplicates. The total protein concentration after the depletion ranged between 1.8 and 2.1 µg/µL with a coefficient of variation of 5.8%.

About 5 µg of total protein from each samples were desalted and trypsinated (1:25, wt/wt trypsin/protein) overnight as previously described.^35^ The tryptic peptides were dried using SpeedVac vacuum concentration system (Savant, Farmingdale, NY).

The peptides were resolved in 0.1% formic acid and approximately 0.25 µg was subjected to nLC-MS/MS analysis. The samples were blinded and randomized during the LC-MS acquisition. A blank sample that consisted of only running buffer was run between every 5 samples. Peptides were separated by reverse phase chromatography on an EASY-nLC II (Thermo Scientific, Waltham, MA), and automated online analyses were performed by a data-dependent acquisition method using LTQ Orbitrap Velos Pro hybrid mass spectrometer (Thermo Scientific) with a nanoelectrospray source. The chromatography was performed on a C18 column (100 mm × 0.75 µm; Agilent Technologies, Santa Clara, CA). The injection volume was 4 μL corresponding to 0.25 μg of protein, and mobile phases consisted of 0.1% formic acid in water (A) and 0.1% formic acid in acetonitrile (B). Peptides were separated by a two-step gradient of 2% to 30% B for 70 minutes and 30% to 100% B for 50 minutes with a flow rate of 300 nL/min.

Raw files were searched using MaxQuant v. 1.5.8.3 (Max Planck Institute of Biochemistry, Martinsried, Germany) against a Uniprot Human database (downloaded July 7, 2016) with the following parameters: trypsin was used as digestion enzyme; maximum number of missed cleavages 2; fragment ion mass tolerance 0.50 Da; parent ion mass tolerance 5 ppm; fixed modification—carbamidomethylation of cysteine; variable modifications—N-terminal acetylation. Data were filtered at 1% false discovery rate.

### 2.4. Multiplex immunoassay analysis

The concentrations of 71 cytokines, chemokines, and growth factors were analyzed using a U-PLEX assay based on an electrochemiluminescent detection method (Meso Scale Diagnostics, Rockville, MD) according to the manufacturer's recommendations. Data were collected and analyzed using MESO QUICKPLEX SQ 120 instrument equipped with DISCOVERY WORKBENCH data analysis software (Meso Scale Diagnostics). Samples were thawed on the day of analysis, blinded, and were randomly mixed.

### 2.5. Statistics

Differences were tested with nonparametric Mann–Whitney and Wilcoxon paired test using IBM SPSS v. 24.0 (IBM), and a *P*-value <0.05 was considered significant. Descriptive data are presented as mean and SD or median and interquartile range depending on normality of the data and type of scale used.

Multivariate correlation between membership of groups and quantified proteins were analyzed with orthogonal partial least square discriminant analysis (OPLS-DA) using SIMCA-P+ version 13.0 (UMETRICS, Umeå, Sweden). The procedure to compute multivariate correlation models has been described earlier.^2,24^ Briefly, before OPLS-DA analysis, a principle component analysis was created to control for multiple outliers. No detectable multivariate outliers were identified in this study. The OPLS-DA model contains data variables that were mean centred and scaled for unified variance (UV-scaled). The variable influence of projection (VIP value) describes the importance and relevance of each X variable (quantified proteins), pooled over all dimensions, and Y variables (CWP or CON) that include the group of variables that best explain Y. In this study, a VIP >1.0 with a jacked-knifed 95% confidence interval was considered significant.^8,38^ The OPLS-DA model of morning and evening values was performed in a 2-step analysis. First, a premodel was built including all proteins. From this model, proteins with a VIP value > 1.0 including the jacked-knifed 95% confidence interval, not including zero, were further used in a new regression model. For the important variables (ie, VIP > 1.0) and to facilitate the understanding of the multivariate results, we also compared evening and morning values using Wilcoxon paired test. *R*^2^ describes the goodness of fit—the fraction of sum of squares of all the variables explained by a principal component. Q^2^ describes the goodness of prediction—the fraction of the total variation of the variables that can be predicted by a principal component using cross-validation methods. *R*^2^ should not be considerably higher (max difference > 0.2 − 0.3) than Q^2^ to avoid overfitting. SIMCA-P+, in contrast to traditional statistical packages such as SPSS, uses the Nonlinear Iterative Partial Least Squares algorithm (NIPALS algorithm) when compensating for missing data—for variables/scales, max 60% missing data and for subjects, max 50% missing data. The NIPALS algorithm was used for all biochemical analyses (both the proteomics and the multiplex immunoassay analysis), both for the samples obtained in the morning and in the evening.

## 3. Results

### 3.1. Data overview

Anthropometric data are presented in Table 1. Participants included in the study showed no signs of psychological or psychosocial distress. Furthermore, there were no significant differences regarding demographic and psychological parameters between males and females (*P* > 0.05).

**Table 1:**
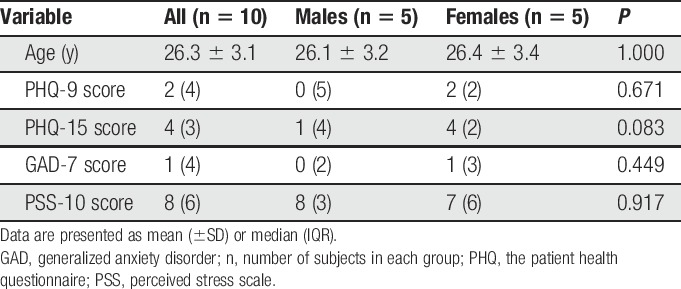
Background data of age and psychological distress of the participants (n = 10).

### 3.2. Proteome patterns in plasma over time

**Figure 1.**
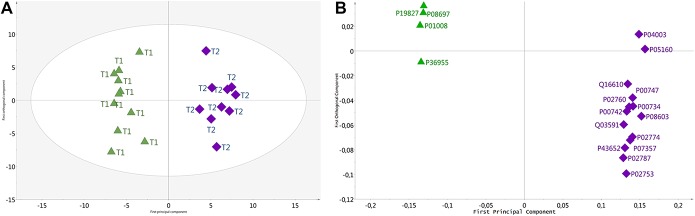
An OPLS-DA (orthogonal partial least square discriminant analysis) model showing separation between plasma protein at time point 1 (morning) and time point 2 (evening). The longitudinal dimension (*y*-axis) shows the interclass discrimination, and the latitudinal dimension (*x*-axis) shows the intraclass discrimination between the groups. Score plot (A) shows the separation between T1 (Triangles) and T2 (diamonds). Loading plot (B) corresponding to significant proteins with a VIP value >1 important for evening (blue square, T2) and morning (green triangle, T1). The proteins are labelled according to their UniProt designation (see supplementary Table 1, available at http://links.lww.com/PR9/A48).

**Figure 2.**
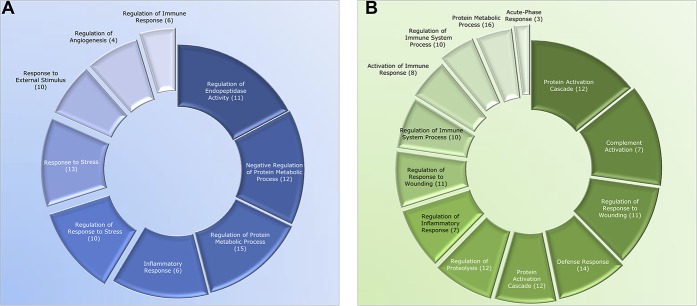
Pie charts classifying the significantly altered proteins based on biological process using STRING pathway analysis. (A) Downregulated proteins. (B) Upregulated proteins in evening vs morning. The names of the genes involved in the different processes are listed in supplementary Table 2A-B (available at http://links.lww.com/PR9/A48).

The proteomic analysis resulted in a number of 279 proteins whose expression levels were detected in at least 50% of the samples in each group. Multivariate statistical analysis showed that there were up to 64 proteins whose expression levels were significantly altered between the plasma samples collected during the morning and afternoon (Fig. 1; and supplementary Table 1, available at http://links.lww.com/PR9/A48). Those proteins together explained 95% (*R*^2^) of the variation and with a prediction of 85% (Q^2^). The coefficient of variation‐analysis of variance revealed that the model was highly significant (*P* < 0.001). The biological processes that the important proteins are involved in are further described in Figure 2. The majority of proteins with VIP >1 were also significant (*P* < 0.05) according to the Wilcoxon paired test. The levels of 34 proteins were increased, and 30 proteins were decreased during the afternoon compared with the morning sample (supplementary Table 1, available at http://links.lww.com/PR9/A48). The increased proteins were involved in biological processes such as the protein activation cascade, complement activation, and response to stress (Fig. 2A; supplementary Table 2A, available at http://links.lww.com/PR9/A48). The decreased proteins were involved in regulation of endopeptidase activity, inflammatory response, and protein metabolic process (Fig. 2B; supplementary Table 2B, available at http://links.lww.com/PR9/A48).

### 3.3. Changes of cytokines/chemokines/growth factors in plasma over time

The multivariate analysis (OPLS-DA) showed no significant separation between morning and evening samples based on the 71 analyzed cytokines/chemokines/growth factors (supplementary Table 3, available at http://links.lww.com/PR9/A48) and we therefore refrained from further conventional statistical analyses (ie, Wilcoxon tests).

## 4. Discussion

Plasma proteomics is a rapidly developing science, depending on many basic developments. The technique is extensively used particularly for the evaluation of protein changes associated with inflammation. The challenge of clinical proteomics studies is to link protein expression profiles to specific disease phenotypes and to find out relevant biomarkers to develop diagnostic and prognostic tools.^33^ Molecular pain biomarkers in plasma have been investigate in several studies that show alterations of several inflammatory proteins in pain patients. Geyer and coauthors studied the interindividual and intraindividual variability in 8 healthy participants and showed that the plasma proteome has much higher interindividual than intraindividual variability.^10^ Other studies have primarily focused on determining the longitudinal changes for protein variants in children and adults.^19,34^ To the best of our knowledge, there is no study that has investigated the effects of sample collection time on inflammatory pain biomarkers. Thus, it is of great relevance to understand the fluctuation in the proteome that normally occurs due to physiological fluctuation in bodily functions.

In the current study, we investigated the diurnal variation of the plasma proteome between morning and evening. The major result was that 64 proteins were identified whose levels were significantly altered between the samples collected in the morning and evening. It was ensured through the clinical examination that the participants were in good health. The inclusion criteria were very strict to reduce the influence of external factors on the outcome. Because the plasma proteome is modified after exercise^25^ and during stress,^33^ participants were asked about the level of physical activity before collection. Psychological factors among participants were also evaluated using validated questionnaires (Table 1). To reduce the influence of psychological distress on the protein expression, participants reporting increased levels of psychological distress were not included. We used a small sample to allow for detection of major changes and to reduce the influence of individual variation and external factors.

Cytokines, chemokines, and growth factors have been linked to different conditions, and some have been shown to be produced and secreted with circadian rhythm. The secretion of proinflammatory cytokines peak (IL-2, IFN-g, TNF-α) during the evening, whereas levels of IL-6 follow a biphasic rhythm.^31^ Differences in median concentrations for several inflammatory substances could be observed between morning and evening samples, but the differences were not significant (supplementary Table 3, available at http://links.lww.com/PR9/A48). However, the expression level of several proteins changed between morning and evening. Coagulation factor XIII B chain was the highest important protein (VIP = 1.8) that contributed to the discrimination between samples collected during the morning and evening. This protein is involved in the fibrinolysis, and a circadian variation of fibrinolytic system in blood has been reported previously.^1^ Those authors reported an increase of the fibrinolytic activity in blood at 6 pm. Thus, the increased level of coagulation factor XIII B chain during the evening in this proteomic study is in the line with previously reported study.

Circadian variation of several proteins involved in innate immunity was also detected. Increased (*P* < 0.05) levels of a number of components of the complement system (complement factor H, C4b-binding protein alpha chain, complement component C8 alpha chain, complement factor H-related protein 1, complement component C7, and C4b-binding protein beta chain), and decreased (*P* < 0.05) levels of other components (complement C2, complement factor B, complement C5, complement factor I, and complement C3), were found in the evening samples compared with the morning samples. The most important complement proteins for the separation between the groups were complement factor H (VIP = 1.77) and C4b-binding protein alpha chain (VIP = 1.71) whose levels were increased during the evening. It has been found that the immune response is depending on the time of the day.^6,17^ For example, plasma levels of C3 and C4 are reported to be influenced by the circadian rhythm and sleep. Poor sleep measures have been associated with increased levels of inflammatory complement components.^14,28^

Few studies have evaluated the plasma proteome in healthy individuals. The advantage of the current study design is that it allows us to study the periodicity of numerous proteins simultaneously, whereas other studies usually have focused on isolated single proteins. The findings for this explorative study should, however, be interpreted within the context of certain limitations. For instance, the study was performed in healthy young adults and included a small number of participants; different age groups were not taken into consideration because of the possibility of age variability. Moreover, the number of men and women were too few to investigate sex differences. Finally, the absence of multiple samples collected during the day and night may have prohibited observations of some important fluctuations.

In conclusion, the results demonstrate that the plasma proteome express fluctuation over time in healthy individuals, which highlights the importance of standardisation in timing of sample collection particularly for the assessment of pain biomarker studies. Larger study cohorts with multiple samples collected frequently during 24-hour period are required to validate the results.

## Disclosures

The authors have no conflict of interest to declare.

The research was financially supported by the Swedish Research Council (K2009-52P-20943-03-2, 2014-2979, 2018-02470), the Stockholm County Council (SOF project), the Swedish Dental Society, ALF grants at Region Östergötland, and AFA Insurance (140341) The funders had no role in the study design, data collection and analysis, decision to publish, or preparation of the manuscript.

## Supplementary Material

SUPPLEMENTARY MATERIAL

## Appendix A. Supplemental digital content

Supplemental digital content associated with this article can be found online at http://links.lww.com/PR9/A48.
